# Using Artificial Intelligence–Based Technologies for the Early Detection of Behavioral and Psychological Symptoms of Dementia: Scoping Review

**DOI:** 10.2196/76074

**Published:** 2025-10-21

**Authors:** Sofia Fernandes, Joëlle Rosselet Amoussou, Carla Gomes da Rocha, Elodie Perruchoud, Armin von Gunten, Cédric Mabire, Henk Verloo

**Affiliations:** 1 School of Health Sciences, University of Applied Sciences and Arts Western Switzerland (HES-SO) Valais-Wallis, Chemin de l'Agasse 5, Sion, 1950, Switzerland, 41 58 606 86 17; 2 Les Maisons de la Providence Nursing Home, Montagnier, Switzerland; 3 Lausanne University Hospital and University of Lausanne Faculty of Biology and Medicine, Institute of Higher Education and Research in Healthcare, Lausanne, Switzerland; 4 Medical Library-Cery, Lausanne University Hospital and University of Lausanne, Site de Cery, Prilly, Switzerland; 5 Service of Old Age Psychiatry, Lausanne University Hospital and University of Lausanne, Lausanne, Switzerland

**Keywords:** artificial intelligence, behavioral and psychological symptoms of dementia, early detection, older adults, scoping review

## Abstract

**Background:**

People with dementia commonly display behavioral and psychological symptoms, which have multiple negative consequences. Artificial intelligence–based technologies (AITs) have the potential to support earlier detection of the behavioral and psychological symptoms of dementia (BPSD). The recent surge of interest in this topic underscores the need to comprehensively examine the existing evidence.

**Objective:**

This scoping review aimed to identify and summarize the types and uses of AITs currently used for the early detection of BPSD among people diagnosed with the disease. We also examined which health care professionals were involved, nursing involvement and experience, the care settings in which these technologies are used, and the characteristics of the BPSD that were assessed.

**Methods:**

Our scoping review was conducted in accordance with the Joanna Briggs Institute manual for scoping reviews. Searches were conducted in March 2025 in the following bibliographic databases: MEDLINE ALL Ovid, Embase, APA PsycINFO Ovid, CINAHL EBSCO, Web of Science Core Collection, the Cochrane Library Wiley, and ProQuest Dissertations and Theses A&I. Additional searches were performed using citation tracking strategies and by consulting the Association for Computing Machinery Digital Library. Eligible studies included primary research involving people with dementia and examining the use of AITs for the detection of BPSD in real-world care settings.

**Results:**

After screening 3670 articles for eligibility, the review includes 12 studies. The studies retained were conducted between 2012 and 2025 in 5 countries and encompassed a range of care settings. The AITs used were predominantly based on classic machine learning approaches and used information from environmental sensors, wearable devices, and data recording systems. These studies primarily assessed behavioral and physiological parameters and focused specifically on symptoms, such as agitation and aggression. None of the retained studies explored nurses’ roles or their specific skills in using these technologies.

**Conclusions:**

The use of AITs for managing BPSD represents an emerging field of research offering novel opportunities to enhance their detection in various health care contexts. We recommended that nurses be actively engaged in developing and assessing these technologies. Future research should prioritize investigations into how effective AITs are across diverse populations, whether they can have a long-term impact on managing BPSD, and whether they can improve the quality of life of patients and caregivers.

## Introduction

### Background

The growing prevalence of dementia is a major global concern [1]. As the disease progresses, cognitive decline is often accompanied by the behavioral and psychological symptoms of dementia (BPSD) [2,3]. The International Psychogeriatric Association defines the BPSD as disturbances in perception, thought content, mood, or behavior [4]. These symptoms can be grouped into two subtypes: (1) behavioral symptoms, which are typically observed directly (agitation, verbal or physical aggression, wandering, sexual disinhibition, repetitive behavior, catastrophic reactions, sundowning, sleep problems, sleeplessness, and apathy), and (2) psychological symptoms, which are usually assessed through interviews with patients and relatives (psychosis, delusions, hallucinations, misidentifications, mood changes, anxiety, restlessness, and depression) [2,4]. BPSD has a deleterious impact on the quality of life of people with dementia, accelerating cognitive decline, reducing life expectancy, and precipitating hospitalizations and early placement in nursing homes [5,6]. Managing patients’ BPSD often requires constant vigilance, leading to heightened stress and depressive symptoms among informal caregivers. Furthermore, indirect costs, including medical consultations, nonreimbursed medication, home adaptations, and reduced professional activity, can lead to substantial socioeconomic burdens [7-9]. For health care professionals, BPSD can increase workloads, stress, and the risk of professional burnout, which may reduce the quality of the care provided [10,11]. Regarding the health care system overall, BPSD leads to higher costs due to more consultations, hospitalizations, and prescriptions of psychotropic drugs [12].

The etiopathogeneses of BPSD are complex and multifactorial, influenced by biopsychosocial and environmental factors, and vary considerably in frequency and intensity between individuals [13,14]. Their early detection through the identification of subtle behavioral and psychological changes (eg, apathy, anxiety, sleep problems, irritability, and social withdrawal), before any formal clinical intervention, is of major clinical importance [15,16]. Early identification enables proactive management and personalized interventions, thereby helping to mitigate the symptoms’ deleterious impact. However, early detection is challenging as early signs are often transient, underrecognized, and misinterpreted as normal aging or personality changes [15,16]. Furthermore, standard detection tools, such as the Neuropsychiatric Inventory, the Alzheimer’s Disease Behavioral Pathology Assessment Scale, or the Cohen-Mansfield Agitation Inventory, can be time-consuming, and health care professionals do not always perceive them to be precise or practical [11,14,17]. These challenges are exacerbated by growing demands for care and persistent shortages of qualified health care staff [18,19]. New health care technologies offer promising solutions to these challenges [20,21].

Recent technological developments have focused on artificial intelligence–based technologies (AITs), which combine artificial intelligence (AI) with remote sensors, robotics, and decision support algorithms [21-24]. AI encompasses a wide range of techniques and methodologies designed to enable computer systems to mimic human cognitive functions, such as learning, problem-solving, and decision-making [24]. When combined with critical thinking and human judgment, AI can optimize clinical reasoning by accelerating and refining assessment, anticipation, the synthesis of multiple data sources, and knowledge generation [25]. In health care, AI is already applied in diagnosis and screening, treatment personalization, patient monitoring, robot-assisted surgery, and health system management [22]. In dementia care, current AITs include monitoring systems, social robots, and daily living assistance tools, primarily involving wearable and environmental sensors [26-28]. These technologies have shown positive impacts on depression, quality of life, social engagement, cognitive function, and patient monitoring [26-28]. They have also demonstrated good performance in health monitoring and dementia screening [26-28].

Management of BPSD requires a multidisciplinary team, with nurses playing a key role in the early detection of symptoms, thanks to their continuous proximity with patients [4]. However, due to the complexity of these symptoms and the diversity of beliefs and conceptualizations regarding BPSD, it is not uncommon for nurses’ clinical judgments to be based on intuition and their standard practice rather than on a clear, standardized methodology [29,30]. This underscores the potential for introducing AITs to support clinical judgment and thus enhance the quality of nursing care for people with dementia. Registered and advanced practice nurses have been actively helping to develop and implement AITs in health care [31-33]. They engage in activities including problem-solving, defining contextual needs and formulating priorities, idea generation, finding concrete solutions, and determining end user satisfaction [31-33].

While previous reviews have examined the use of AITs in the care of patients with dementia and BPSD, their scope differed from this work [34-38]. Husebo et al [37] focused on treatment monitoring, and Jao et al [35] emphasized model performance without mapping contexts of use. AI has evolved rapidly since 2020, and it is likely that new solutions, not covered in these reviews, have now emerged. More recently, other authors have addressed the use of AITs in the general care of those with dementia and BPSD, combining prediction, assessment, and treatment, but without a specific focus on early detection [34,38]. Similarly, Shaik et al [36] reviewed remote sensing technologies and care frameworks without focusing on the application of AITs for the early detection of the BPSD. Our preliminary literature searches identified no completed or ongoing scoping reviews on the topic. Given the rapid evolution of AITs and their growing integration into dementia care, an updated and targeted synthesis of the literature is necessary.

### Aim and Review Questions

This scoping review aimed to identify and summarize the different types of AITs currently available for the early detection of BPSD among older adults. It also sought to examine the functional uses of those technologies, the health care professionals involved, nursing involvement and experience with AITs, the care setting in which they are used, and the characteristics of the BPSD they assess. Our research questions were developed following the population, concept, and context framework. First, what type of AITs are currently available for the early detection of BPSD among people with dementia? Second, what are the functional uses of the AITs involved in the early detection of BPSD? Third, which health care professionals are involved in the use and implementation of AITs for the early detection of BPSD? Fourth, in which care settings are AITs used for the early detection of BPSD? Which specific characteristics of BPSD do AITs assess? Finally, what are nurses’ specific roles, responsibilities, and experiences regarding the use of AITs for the early detection of BPSD?

## Methods

### Protocol and Registration

This scoping review was conducted in accordance with the Joanna Briggs Institute manual for scoping reviews [39]. The PRISMA-ScR (Preferred Reporting Items for Systematic Reviews and Meta-Analyses extension for Scoping Reviews; checklist provided in Checklist 1) standards were followed for reporting [40]. The scoping review’s protocol was registered at the Open Science Framework [41].

### Eligibility Criteria

The eligibility criteria for studies with the potential to be assessed in this review were defined based on their population, concepts, and types of sources. The inclusion and exclusion criteria are detailed in Table 1.

**Table 1.** Scoping review inclusion and exclusion criteria.

Criteria	Inclusion criteria	Exclusion criteria
Participants	Studies involving people aged 65 y and older with any dementia (eg, Alzheimer disease, frontotemporal dementia, vascular dementia, and Parkinson dementia).	Studies involving individuals with mental disorders other than dementia (eg, mild cognitive impairment, depression, delirium, schizophrenia, and bipolar disorder).
Concept		
Artificial intelligence: techniques and methodologies designed to enable computer systems to mimic human cognitive functions, such as learning, problem-solving, and decision-making [24]. Other terms: “computational intelligence,” “computer reasoning,” “machine learning,” and “machine intelligence.”	Studies involving all types of devices and software based on AITs^a^ to screen, detect, and assess clinical BPSD^b^.	Studies focusing on AITs intended for the prediction or management of symptoms or diseases (eg, type and stage of dementia, psychiatric disorders, chronic illnesses, and acute conditions).
BPSD: Disturbances in perception, thought content, mood, or behavior [4].	Studies involving all types of clinical BPSD: agitation, aggression, verbal aggression, physical aggression, psychosis, delusions, hallucinations, misidentifications, repetitive behavior, wandering, sundowning, restlessness, catastrophic reactions, sexual disinhibition, mood changes, depression, apathy, anxiety, sleep problems, and sleeplessness [4].	Studies that did not use scales rating BPSD to report psychological symptoms.
Context		
(1) Acute care: Immediate and intensive treatment services provided within the health care system to address severe, sudden and often unforeseen health episodes: emergency medicine, trauma services, prehospital emergency response, acute surgical care, critical care units, urgent care, and short-term stabilization of patients in an inpatient setting [42]. (2) Community care: social support services and programs designed to empower individuals with specific needs—including those with intellectual disabilities, mental health challenges, and older adults—to lead independent lives and actively engage in their communities [43]. (3) Long-term care: support services aimed at addressing the health and personal care needs of individuals who are unable to manage daily activities independently: home-based care, community care and residential care [44].	Studies involving acute care, community care, or long-term care from any country.	Studies involving virtual environments.
Types of sources	Experimental and quasi-experimental studies; Analytical and descriptive observational studies; Qualitative and mixed methods studies.	All types of reviews: narrative reviews, integrative reviews, scoping reviews, systematic reviews, umbrella reviews, and bibliometric analyses; Opinion letters; Commentaries; Books and book chapters.

a AIT: artificial intelligence–based technology.

b BPSD: behavioral and psychological symptoms of dementia.

### Information Sources and Search Strategy

In March 2025, we conducted a literature search of the following bibliographic databases in collaboration with a medical librarian (JRA): MEDLINE ALL Ovid, Embase, APA PsycInfo Ovid, CINAHL EBSCO, Web of Science Core Collection, the Cochrane Database of Systematic Reviews Wiley, the Cochrane Central Register of Controlled Trials Wiley, and ProQuest Dissertations and Theses A&I. No language or date restrictions were applied. The final search strategies were peer-reviewed by a second information specialist and are documented in Multimedia Appendix 1, detailing the search syntax, keywords, and index terms used. Using the studies that were retained, we performed a backward and forward citation search using Citationchaser [45] (SF). Further research was carried out in the Association for Computing Machinery Digital Library (SF).

### Study or Source of Evidence Selection

After the search, a medical librarian (JRA) imported all the references identified into EndNote 20 (Clarivate Analytics), and duplicate references from the databases were removed using Deduklick (Risklick AG) [46]. The references were then imported into the Rayyan interface, and 2 reviewers (SF and CGdR) independently screened the titles and abstracts according to the established inclusion criteria. All the potentially relevant sources were retrieved in full and imported into a database. Two reviewers (SF and EP) then independently assessed the full texts of the references retained to ensure they met the inclusion criteria. The rationales for excluding references at the full-text review stage were documented. Disagreements between the reviewers during this selection process were discussed and resolved with the assistance of a third reviewer (HV). Due to resource constraints, the selection of references identified through citation tracking was conducted by a single reviewer (SF). The results are presented in a PRISMA-ScR flow diagram [47].

### Data Extraction

Data from the references retained for the scoping review were extracted by a single reviewer (SF), and a random 10% subset of the full-text articles included was verified by a second reviewer (HV) to ensure accuracy and completeness [48]. The second reviewer cross-checked the completed data extraction tool’s results against the corresponding full-text articles. Data were extracted using a data extraction tool developed in accordance with the study’s objectives and search questions. The data extracted included the year of publication, country of origin, study characteristics (design, objectives, sample size, health care setting, and health care professionals), participant characteristics (age, sex, and dementia type and severity), specific types of the AITs (sensors, social robots, and decision support systems), delivery methods for these AITs (remote monitoring platforms and mobile apps), the functional purposes of these AITs, the AI techniques applied, characteristics of the BPSD assessed, the traditional instruments used for detecting BPSD, nurses’ involvement and experience, and key findings relevant to the review questions (eg, effectiveness, usability, feasibility, users’ satisfaction with AITs, users’ perspectives about AITs, and barriers and facilitators to adopting AITs). When necessary, study authors were contacted to request missing or additional data.

### Data Analysis and Presentation

We use a narrative approach to present the extracted and analyzed evidence in the different sections below [39]. The sections include (1) the characteristics of the included studies, (2) the types and uses of AITs, (3) the characteristics of the BPSD detected, and (4) the involvement and experience of nurses. The types of AITs were analyzed and categorized according to the definitions proposed by Zhang and Lu [49], which distinguish the different drivers and technologies of AI. Tables, charts, and diagrams are used to summarize and make the evidence more readable.

## Results

**Figure 1.**
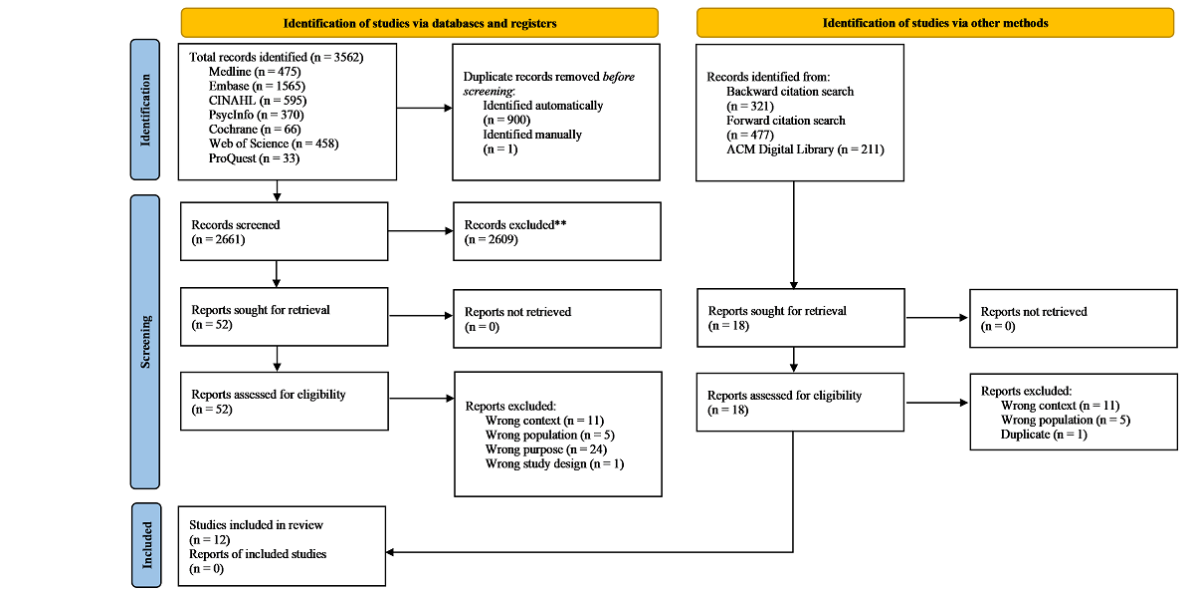
PRISMA (Preferred Reporting Items for Systematic Reviews and Meta-Analyses) 2020 flow diagram (source: Page et al [47]).

Our bibliographic database searches identified 3562 publications. After removing 901 duplicate records, 2661 publications underwent title and abstract screening. Of these, 2609 were excluded for not meeting our inclusion criteria, and 41 of the 52 full-text articles assessed for eligibility were excluded. Following the recommendation of an expert, 18 full-text articles were found using citation searching and a search of the Association for Computing Machinery Digital Library; however, after assessing them for eligibility, 17 were excluded. The scoping review finally retained 12 articles [50-61] (Figure 1).

### Study Characteristics

The 12 studies reviewed came from 5 countries and were published from 2012‐25. Over half of the studies were published in the last 5 years (2020‐2025) [50-56]. They included 3 single case studies [53,57,58], 2 studies using quasi-experimental approaches [52,59], 1 randomized controlled trial [60], 1 correlational study [61], 1 exploratory observational study [54], 3 retrospective longitudinal studies [50,55,56], and 1 qualitative study [51]. Furthermore, 3 publications examined acute care settings [52,57,61], 6 examined community care settings [50,54,55,58-60], and 3 examined long-term care settings [51,53,56].

The study participants included groups of older adults with mean ages ranging from 78.8 to 84 years old and presenting with mild to advanced dementia. Although 1 study [51] did not involve participants with a confirmed diagnosis of dementia, it was retained due to its focus on the implementation of a behavioral disturbance detection system in long-term care settings, which predominantly serve people with dementia. As such, it provided valuable contextual insights relevant to integrating such technologies into dementia care. Most of the health care professionals included in the studies were nurses [51-54,56,57], followed by physicians [53,56,57], care workers [51,56], a rehabilitation assistant [51,56], an occupational therapist [51,56], and a physiotherapist [51,56]. Table 2 describes the main characteristics of the studies retained.

**Table 2.** Characteristics of the studies retained (n=12).

Study, country	Design (aim)	Sample size (N)	Health care setting	Health care professionals involved	Participants (age, sex, dementia type, and severity)
Bafaloukou et al [50], United Kingdom	Retrospective longitudinal study (develop a machine learning framework able to identify episodes of agitation).	63	Community care (home)	—^a^	Age: N/P^b^, sex: female (n=22), male (n=41), dementia: Alzheimer, vascular frontotemporal, Parkinson
Bourennane et al [57], France	Single case study (describe experiments to detect deviations in a patient’s behavioral observations using a multi-sensor).	1	Acute care (Alzheimer’s care unit)	Physicians and nurses	Age: mean 84 y, sex: female, dementia: Alzheimer
Choukou et al [51], Canada	Qualitative case study (explore the perceptions of healthcare professionals and family members about the potential for introducing a behavioral disturbances detector in long-term care homes).	8 health care professionals and 1 proxy	Long-term care (tertiary care facility)	Nurses, care workers, rehabilitation assistants, occupational therapists, and a physiotherapist	Age: N/P, sex: female (n=9), dementia: N/P
Enshaeifar et al [59], United Kingdom	Quasi-experimental, interrupted-time series (detect patterns directly correlated with agitation, irritation, and aggression in people with dementia).	12	Community care (home)	—	Age: N/P, sex: N/P, dementia: mild to moderate
Iaboni et al [52], Canada	Quasi-experimental, interrupted-time series (develop personalized machine learning models capable of detecting individual patterns in BPSD^c^).	17	Acute care (specialist dementia unit)	Nurses	Age: mean 78.9 (SD 8.9) y, sex: female (n=10), dementia: advanced
Kim et al [53], South Korea	Single case study (detect a dementia patient’s abnormal behavior using sensors and developing a machine-learning-based system).	1	Long-term care (nursing homes)	Physicians and nurses	—
König et al [61], France	Correlational (investigate whether automatic speech analysis could be used to characterize and detect apathy).	60	Acute care (hospital memory clinic)	—	Age female with apathy: mean 79.50 (SD 5.86) y, age male with apathy: mean 79.58 (SD 5.45) y, dementia: N/P
Lee et al [54], South Korea	Exploratory observational study (investigate a predictive model for sleep efficiency and its associated features among older adults living with dementia in their own homes).	69	Community care (home)	Nurses	Age sleep efficiency >85%: mean 80.15 (6.11) y, Age sleep efficiency <85%: mean 80.64 (4.63) y, sex: N/P, dementia: N/P
Lotfi et al [58], United Kingdom	Single case study (design unobtrusive activities in a daily living monitoring system for identifying and predicting abnormal behavior).	1	Community care (home)	—	Age: N/P, sex: female, dementia: N/P
Palermo et al [55], United Kingdom	Retrospective longitudinal study (identify episodes of agitation among people living with dementia using a deep recurrent model applied to continuous in-home monitoring data).	Total=46 (33 for training, 8 for validation, and 5 for testing)	Community care (home)	—	Age: mean 82.6 (SD 7.2) y, sex: N/P, dementia: Alzheimer, frontotemporal, vascular, Parkinson
Rostill et al [60], United Kingdom	Randomized control trial, parallel groups (test the technology integrated health management system in a real-world setting).	204 patients and 204 carers	Community care (home)	—	Age: N/P, sex: N/P, dementia: mild to moderate
Zhu et al [56], Australia	Retrospective longitudinal study (determine the prevalence of agitated behaviors among people with dementia using natural language processing).	3528	Long-term care (nursing homes)	Nurses, care workers, and other health care professionals (eg, physicians and therapists)	Age: N/P, sex: female (n=2267), male (n=1261), dementia: N/P

a Not applicable.

b N/P: not provided.

c BPSD: behavioral and psychological symptoms of dementia.

### AITs and Their Use

#### Data Collection

Most of the studies adopted a multimodal input approach, combining 2 or more data types. In total, 6 studies reported on combinations of environmental sensors and wearable devices [50,53-55,57,59,60] and 1 study used wearable devices with video cameras [52]; 2 studies relied on signal data, referring to continuous, time-series measurements, captured through voice recorders or environmental sensors [58,61]. Voice recorders were not classified as wearable devices as they were set in fixed positions during structured interviews rather than being worn continuously for passive data collection. One study used structured data derived from an electronic health care records system [56]. The studies used a variety of environmental sensors, including passive infrared sensors or motion detectors, door and window entry point sensors, electricity power usage sensors, and bed and sofa pressure sensors. Wearable devices included wristbands, smartwatches, sweat patches, and GPS trackers integrated into textiles or attached to the body. These devices were equipped with sensors collecting motion data via accelerometers, monitored sleep patterns through actigraphy, and recorded other structured data, such as blood volume pulse, electrodermal activity, transpiration, and skin temperature. Smart thermometers and smart scales were used to collect physiological data, including body temperature, body composition, and heart rate. Furthermore, 4 studies reported using wireless technology systems to communicate between the various devices [53,57,58,60].

#### AI Learning Processes and Approaches

Supervised learning processes were used in 7 studies in which models were trained on labeled datasets to predict specific behavioral outcomes [50,52-55,58,61]; 2 studies used unsupervised learning approaches aiming to discover patterns or clusters within unlabeled data [56,59], while 1 study adopted a hybrid process combining both supervised and unsupervised methods [57]. Most of the studies relied on machine learning techniques, including algorithms such as random forest, logistic regression, k-nearest neighbors, decision trees, support vector machines, LightGBM, and CatBoost [50,52-54,56,57,59-61]. Only 2 studies applied deep learning methods, specifically recurrent neural networks and long short-term memory networks [55,58]. Table 3 provides an overview of the main data sources, AI techniques, learning processes, and AI approaches reported in the studies selected.

**Table 3.** Principal methods and data sources of the artificial intelligence–based technologies used.

Study	Data sources (input)	AI^a^ technique	Learning process		AI approach	
			Supervised	Unsupervised	Machine learning^b^	Deep learning^c^
Bafaloukou et al [50]	Multimodal signal	Light gradient-boosting machine	✓	—^d^	✓	—
Bourennane et al [57]	Multimodal signal	K-means, k-nearest neighbors	✓	✓	✓	—
Enshaeifar et al [59]	Multimodal signal	Markov chains	—	✓	✓	—
Iaboni et al [52]	Multimodal signal	Random forest	✓	—	✓	—
Kim et al [53]	Multimodal signal	k-nearest neighbors, random forest, logistic regression, decision tree, support vector machine, multilayer perceptron	✓	—	✓	—
König et al [61]	Signal	Logistic regression	✓	—	✓	—
Lee et al [54]	Multimodal signal	CatBoost	✓	—	✓	—
Lotfi et al [58]	Signal	Recurrent neural networks	✓	—	—	✓
Palermo et al [55]	Multimodal signal	Long short-term memory	✓	—	—	✓
Rostill et al [60]	Multimodal signal	N/P^e^	N/P	N/P	✓	—
Zhu et al [56]	Structured data	Computerized natural language processing	—	✓	✓	—

a AI: artificial intelligence.

b Machine learning is a set of algorithms that learn patterns from data using manually engineered features, often applied to structured datasets [24].

c Deep learning uses artificial neural networks to automatically learn hierarchical feature representations directly from raw data [24].

d Not applicable.

e N/P: not provided.

#### Data Availability and Visualization

Of the total, 2 studies included used cloud-based systems to retrieve data from devices and transmit them to AI-driven analytical platforms [53,60], while 1 study used the Zigbee communication protocol to transmit data [57]. Furthermore, 5 of the studies retained made their data derived from AI available and visualized it on secure web portals [55,57-60]. In comparison, 1 study used a mobile health app [53]. In addition, 6 studies described the use of AITs with alert systems triggered by the detection of abnormal behavior [50,53,55,57,59,60]; 4 studies reported using AITs to identify abnormal behavior without an alert system [52,56,58,61]. The studies retained in our analysis defined abnormal behavior heterogeneously, and 4 studies defined it as a deviation from the established norm in each participant’s profile [53,57-59]. These deviations were characterized by different behavioral features, and their nature varied across studies, with 2 focusing on movement in specific rooms or living areas [57,58], 2 focusing on agitation [57,59], and 1 focusing on repetitive questions, behaviors, and sleep profiles [53]. None of these 4 studies provided any specifications regarding the variance defined by the norm. The principal elements of the interventional studies are outlined in Table 4.

**Table 4.** Principal elements of the interventional studies.

Study	Intervention duration	Device used	Intervention description
Bafaloukou et al [50]	27 mo	Passive infrared sensors and door and kitchen appliance sensors Bed mattresses	Weekly behavioral monitoring questionnaire to report the presence or absence of participants’ behavioral symptoms. Traffic light system (interactive interface), grouping estimates of the probability of agitation produced by the model.
Bourennane et al [57]	30 d	Wearable tag with an inbuilt GPS tracker Infrared and pressure detectors in bed mattresses	Wireless communication technology system to relay data to a central computer in the nursing staff area. Abnormal behavior: hyperactivity, deviations from the standard means^a^ for patient movements through common living areas by time of day and for agitation profiles from the past 30 d. Alerts sent via SMS text messages to caregivers’ mobile phones.
Enshaeifar et al [59]	6 mo	Passive sensors: 2 passive infrared sensors, 4 motion sensors, 2 pressure sensors, 1 door sensor, and 1 central energy consumption monitoring device Bluetooth-enabled medical devices	Anomaly detection of outlying values^a^ for agitation, irritation, and aggression during daily activities. Alerts were generated on a secure web portal and verified by the clinical team by contacting the caregiver.
Iaboni et al [52]	8 wk	Wristband (Empatica E4) Video cameras installed in common living areas	Customized machine learning models to classify the presence of behavioral symptoms by type (aggression and agitation).
Kim et al [53]	30 d	Smartwatch Lifelog sensors attached to assorted pieces of furniture	Wireless communication technology system interacting between a smartwatch and lifelog sensors. Abnormal behavior: repetitive questions or behaviors and sleep disturbances. Multilayer perceptron machine learning model determined the presence or absence of abnormal behavior. Alerts were transmitted to the smartphones of physicians and nurses via an app.
Lee et al [54]	14 d	Wristband (Actiwatch2 [Respironics, Inc]) Sweat patches (PharmChek [SCRAM Systems])	Sleep efficacy analysis: actigraphy for sleep and physical activity, sweat patch for cytokines. Machine learning analysis to select the sleep efficiency prediction model and exploration of its top 10 associated features. Categorical boosting machine learning algorithm based on decision trees.
Lotfi et al [58]	18 mo	JustChecking wireless motion sensor in the kitchen, bedroom, bathroom, and living room Door entry point sensors in the front and back doors	Abnormal behavior: deviations from the standard mean^a^ for the length of time spent in a specific area of the house. Recurrent neural networks to analyze data with results transmitted to caregivers via a secure web interface.
Palermo et al [55]	27 mo	Motion and tracking sensors (multiple doors, hallway, kitchen, and other rooms) Home appliance use, smart plugs (kitchen appliances, television, and other commonly used devices) Wearable device Smart temporal thermometer Smart scale	Digital platform to integrate and analyze data. Data labeled as true (episode of agitation validated by a clinical monitoring team) or false (not an episode of agitation when the monitoring team contacted participants). Recurrent neural networks are used to analyze the risk of episodes of agitation.
Rostill et al [60]	6 mo	Sensors Vital signs monitors GPS tracker Gateway device	Machine learning algorithm to analyze data. Alerts generated and prioritized on a digital dashboard and followed up by a centralized monitoring team overseen by clinicians. Alert follow-up process: contact caregivers and, if necessary, liaise with other services (social care teams, Alzheimer’s Society, and emergency services).

a Defined threshold not available.

#### AI Model Performance

Of the total, 8 studies reported that their AI model performed well in identifying and classifying distinct types of BPSD [50,52-56,59,61]. The performance metrics used included accuracy, precision, recall, *F*_1_-score, and area under the curve, as they are commonly defined in the literature [62]: accuracy refers to the proportion of correctly classified instances among all instances; precision measures the proportion of true positives among all positive predictions, while recall reflects the proportion of true positives correctly identified among all actual positives; the *F*_1_-score is the harmonic mean of precision and recall, balancing both metrics, the area under the curve indicates the model’s ability to distinguish between classes across all thresholds. The parameters used and their corresponding scores are described in Table 5.

**Table 5.** Performance of the artificial intelligence models used in the retained studies.

Study	Model or algorithm	Performance metrics, mean (SD)				
		Accuracy	Precision	Recall	*F*_1_-score	AUC^a^
Bafaloukou et al [50]	Gradient boosting algorithm	mean 71.3 (SD 7.21)	mean 71.81 (SD 7.94)	N/P^b^	mean 71.12 (SD 7.28)	mean 77.63 (SD 6.59)
Enshaeifar et al [59]	Hierarchical fusion algorithm	N/P	0.81	0.83	0.80	N/P
Iaboni et al [52]	Interpretable models	N/P	N/P	N/P	N/P	0.87
Kim et al [53]	Multilayer perceptron model	0.99	1.00	1.00	1.00	N/P
König et al [61]	Logistic regression models	N/P	N/P	N/P	N/P	0.88 for men and 0.77 for women
Lee et al [54]	Categorical boosting algorithm	0.96	0.87	0.77	0.82	0.99
Palermo et al [55]	Recurrent neural networks model	N/P	0.27	0.79	0.37	N/P
Zhu et al [56]	Rule-based NLP^c^ model	N/P	N/P	N/P	N/P	0.89

a AUC: area under the curve.

b N/P: not provided.

c NLP: natural language processing.

#### The Adoption of AITs

In total, 2 studies reported that the participating health care professionals found AITs useful in helping them understand patients’ health statuses [51,60]; 1 study reported that its participants perceived the use of AITs to be feasible [54]. Another study documented how the majority of its participants, both caregivers and patients, accepted and would have given a favorable recommendation on using the AIT under evaluation [60]. These positive perceptions were primarily linked to the devices’ ease of use and the involvement of a clinical monitoring team to interpret the data generated by the system. Indeed, the few nonendorsements of that AIT were attributed to technical issues, user-perceived difficulties with device use, and the perceived inconvenience associated with the routine monitoring of vital signs. One study identified forgetfulness, refusal, and a lack of cooperation as the primary factors contributing to patients not adhering to their wearable device protocol [54]. Technical problems, such as battery discharge, software errors, device malfunction, and detection failures were also reported as contributing factors [54]. One study referenced future potential AIT users’ perceived requirements for successfully incorporating AITs into clinical environments [51]. These included the necessity for training, the standardized use of technical devices and giving priority access to them to nursing and care staff. Furthermore, that study indicated that those potential AIT users had concerns about data confidentiality and the costs associated with AITs [51].

### Characteristics of the BPSD Detected

The studies retained for analysis reported their assessments of specific behavioral and physiological parameters acting as surrogate markers of BPSD. Regarding behavioral parameters, 6 studies assessed motion [50,52,53,55,57,60], 4 assessed activities of daily living [53,55,56,58], 1 assessed sleep efficiency [54], and 1 assessed speech quality [61]. Regarding physiological signs, 5 studies assessed vital and biological signs (blood pressure, heart rate, body temperature, blood oxygenation, weight, and hydration) [50,52,55,59,60]. One study measured electrodermal activity [52], while another study examined patients’ levels of cytokines, such as interleukin-6 and interleukin-10 [54]. One study examined environmental factors associated with BPSD, such as indoor light levels and ambient temperature [50].

Furthermore, 2 studies focused on detecting the 3 specific BPSD, that is, agitation, irritation, and aggression [59,60]; 3 studies examined agitation alone [50,55,56], while another concentrated on detecting agitation and aggression [52]. In addition, 1 study investigated apathy as a distinct form of BPSD [61], and another concentrated on sleep efficacy [54].

Regarding the use of specific scales for detecting and measuring BPSD, 4 studies used the Neuropsychiatric Inventory [50,52,54,61], and another combined the Neuropsychiatric Inventory with the Apathy Inventory [61].

One study indicated that biological parameters, including blood pressure, blood oxygen, heart rate, body temperature, and hydration, were the most significant markers for detecting agitation, irritability, and aggression [60]. Another study identified heart rate and body temperature as the principal markers for verbal aggression, while an accelerometer reading was the most significant marker for physical aggression [52]. One study identified respiratory rate, number of nocturnal awakenings, and the indoor light level as the most important characteristics for identifying agitation [50]. Table 6 summarizes this information.

**Table 6.** Characteristics of the behavioral and psychological symptoms of dementia found in the studies retained for analysis.

Sign of BPSD^a^	Types of BPSD		
	Agitation or aggression	Apathy	Sleep problems
Behavioral	Sleep Daily activity pattern Sequence of daily activities Movement activity patterns	Paralinguistic markers	Physical activity Percentage of time awake Total sleep duration Daytime sleep minutes Nocturnal sleep minutes Sleep regularity
Physiological	Blood pressure Heart rate Blood volume pulses Oxygen saturation Electrodermal activity Body temperature Weight Hydration	None	Interleukin-6 Interleukin-10

a BPSD: behavioral and psychological symptoms of dementia.

### Nurses’ Involvement and Experience

Only 3 of the studies retained clearly described nurses’ roles in identifying BPSD [51,52,54]. One documented their participation in data collection, specifically documenting episodes of BPSD [52]. Another study described their involvement as participants in a group of health care professionals and the exploration of their perceptions of the potential use of AITs in clinical practice [51]. In research contexts, nurses were part of the research team in 1 study [52] and led the design and implementation of another [54]. None of the retained studies reported on the extent of nurses’ knowledge, skills, and experience in the use of AITs.

## Discussion

### Principal Results

This scoping review highlighted how AITs are emerging and being used for the early detection of BPSD, as evidenced by the limited but growing number of publications over the past 5 years (between 2019 and 2025). Despite this limited body of literature, the studies retained were diverse in terms of design, care environment, and health care professional involvement, as well as in terms of their study populations’ characteristics, and this provided a more comprehensive understanding of the various approaches to using AITs in the care of people with dementia.

The studies included in this review encompassed the domains of acute, community, and long-term care and included several types of health professionals, such as physicians, nurses, home care workers, and therapists. Only one of the studies included explored the integration of AITs from an interprofessional perspective, highlighting potential benefits such as improved communication and more proactive care [51]. These findings aligned with previous reviews highlighting AITs’ roles in enabling real-time clinical decision-making and interdisciplinary data sharing [36,38]. However, health care professionals also expressed concerns about how the challenges of implementing AITs might hinder their successful adoption in daily practice, such as workflow disruptions, overreliance on technology, threats to professional identity, costs, and data privacy [51]. These concerns, also reported in previous literature [36,63,64], involve organizational, technological, professional, and cost issues. Identifying these barriers early on is therefore essential to guiding implementation strategies and systems design, thereby enhancing the usability, acceptability, and integration of AITs, particularly in complex interprofessional settings such as dementia care.

Regarding data collection systems and devices, many of the retained studies reported on the combined use of environmental sensors and wearable devices [52,53,55,57,60]. It should be noted that the innovation in these studies does not lie in the use of particular devices or the application of AI, as both have been used before in dementia care settings, particularly to monitor and enhance cognitive function, as well as to track daily activities and physiological parameters [26,28,36,64-66]. Distinctive innovation lies in integrating data collected by these devices with an AI-based analysis specifically aimed at identifying and generating alerts for episodes of BPSD. While similar integrations have been explored in broader smart health monitoring contexts, their targeted use for the early detection of BPSD remains largely underexplored [26,37,38]. These findings are consistent with those reported in previous reviews, which also mentioned mobile apps, text analysis systems, and robots [27,36-38]. For instance, Shaik et al [36] reported on the use of robots capable of monitoring patient behavior. However, none of the studies included in our review used robots specifically for the detection of BPSD, reflecting a broader trend where AITs form parts of psychosocial interventions rather than acting as early detection tools [34].

The variety of connectable devices is particularly interesting, offering health care professionals the flexibility to tailor technologies to care objectives, patients’ preferences, and context-specific needs. Concerning communication systems and data visualization, most of the studies retained used wireless technologies and a secure web portal, reflecting the growing integration of the Internet of Medical Things in health care [53,57-60]. This type of infrastructure enables real-time data transmission and storage, supporting remote monitoring and system interoperability [67]. Using wireless systems facilitates technological integration by reducing the physical constraints that can be particularly relevant for people with dementia, as restrictive or obtrusive devices could cause distress or resistance [68]. However, device acceptability varied across the studies retained, reflecting the mixed findings reported in previous literature [27,36,67]. Acceptability was primarily judged according to ease of use [51,54,60], consistent with previous reviews that also emphasized the importance of simple, intuitive interfaces [27,36]. Regarding device design, 1 study on wearable devices reported their overall feasibility [54]. However, this contrasted with the conclusion by Loveys et al [27], who identified several barriers to acceptance, including discomfort when wearing devices, intrusiveness due to continuous monitoring, and sleep disturbances caused by bed sensors. Another study involving wearable devices similarly reported such inconveniences, particularly a feeling of being constantly monitored [60], in alignment with the barriers described by Loveys et al [27]. Additional concerns included technical issues and forgetting to wear the device [51,60]. These findings are consistent with previous literature [27,36,67] and further reinforce the importance of anticipating such barriers early on in the system design process to improve the user experience and support successful implementation in dementia care settings.

None of the studies included used advanced data storage and sharing systems (such as edge computing) that can improve security and enable the offline functionalities that are important in settings with limited or unreliable internet access, such as in home care or remote regions. While data security remains a major concern in the Internet of Medical Things, AI offers promising support. As Messinis et al [69] stated, machine and deep learning techniques can enhance cybersecurity. However, in the studies reviewed, AI was primarily used for data analysis in research contexts rather than being fully integrated into clinical practice.

Most of the studies retained used classic machine learning to classify or detect BPSD, training models on labeled data to identify predefined behaviors [50,52-55,58,61]. This reflects a broader trend in early-stage research, where annotated datasets are key for model development and validation [35,36].

While some of the studies retained defined abnormal behaviors as deviations from individual baselines, alerts for the occurrence of BPSD were not systematically triggered at fixed thresholds [53,57-59]. Instead, detection often relied on adaptive methods, such as multivariate pattern recognition or unsupervised learning algorithms that adjusted to individual variations over time. These approaches are particularly well-suited to the heterogeneity and fluctuation of BPSD [13,14] and have the added advantage of requiring few or no labeled data, making them suitable for real-time and continuous monitoring [24,70]. In parallel, some studies used expert validation to verify the accuracy or clinical relevance of alerts, either through retrospective review by clinicians [55,57,60] or the integration of rule-based insights during model development [52,53,59]. One study combined supervised and unsupervised techniques to enhance adaptability to individual patterns and clinical relevance, thus supporting a flexible, person-centered approach [57].

The studies retained for review assessed behavioral and physiological parameters as signs of the BPSD, with most being behavioral parameters, including movement and the activities of daily living [50,52-58,60,61]. Regarding specific BPSD, agitation, irritability, aggression, and apathy were the most frequently investigated [50,52,55,56,59-61], with biological parameters and accelerometer data being the most significant markers for detecting them [52,60]. This particular focus may be explained by the clinical urgency and disruptive nature of these symptoms, which place a significant burden on care staff and often require rapid intervention [5,71,72]. Furthermore, these behaviors tend to generate observable and measurable physiological signals, making them more technically accessible to current AITs. These findings were consistent with previous reviews, one of which also highlighted wandering as a distinct type of BPSD commonly targeted by AITs [35,36]. However, this trend underscores the need to assess how effectively AITs can capture the broader spectrum of BPSD, particularly those that are less externally visible, lack distinct physiological markers, and may manifest more gradually over time, such as anxiety, mood-related symptoms, or apathy.

Overall, the algorithms developed across the retained studies demonstrated a good ability to detect the BPSD targeted. This observation aligned with the findings of other reviews, which report the promising detection or classification accuracy of AITs in dementia care contexts [35,36]. However, some limitations must be considered, as they constrain the generalizability of our findings. These include the small sample sizes used in certain studies and the limited occurrence of behavioral events, which may have affected the robustness of algorithm training. Furthermore, although the diversity of study settings and populations represents a strength of the current body of evidence, the heterogeneity in study contexts and their definitions of abnormal behaviors may have limited their comparability and the broader applicability of our findings.

Although nurses represented the majority of the health care professionals included in the studies retained, their knowledge, skills, and experiences of using AITs to detect the BPSD remain undocumented. Given their central role in detecting and managing BPSD, the lack of insight into how nurses interpret algorithmic outputs, respond to alerts, and integrate AITs into their clinical reasoning raises concerns about the clinical relevance, acceptability, and long-term sustainability of such technologies. Only 2 studies included nurses in their research teams, and only one of those was clearly led by nurse researchers [52,54]. The absence of nursing-specific expertise in the development process may result in technologies that are misaligned with real-world care dynamics, increase the risk of misinterpreting AIT-generated alerts, or inadvertently add to workloads rather than reducing them. These findings appear to contradict the existing literature, which emphasizes nurses’ active involvement in developing and implementing AITs in diverse settings and domains of care, including geriatric care [31,33].

### Implications for Practice and Research

Given the lack of studies examining the efficacy of AITs for detecting the BPSD, recommendations regarding their use remain limited and cannot be generalized to clinical practice. Furthermore, the heterogeneity of devices, data types, and analytical approaches hinders the establishment of standards and limits the development of evidence-based recommendations.

Nevertheless, the proliferation of AITs in clinical and care environments is an emergent phenomenon that cannot be overlooked. To ensure that these technologies are properly understood, safely integrated, and clinically meaningful, it is essential to develop core competencies and critical awareness of AITs at an early stage. In parallel, assessing health care professionals’ readiness to provide feedback for training is equally important as it allows educational initiatives to be tailored to existing knowledge, expectations, and perceived needs. This effort should be grounded in an interprofessional perspective, as the implementation and interpretation of AIT outputs typically involve diverse professional disciplines, including nurses, physicians, and allied health staff. Frameworks, such as the conceptual framework for digitally supported communication, coordination, cooperation, and collaboration model, which emphasizes communication, coordination, cooperation, and collaboration, may provide a useful basis for aligning technological solutions with clinical goals, care contexts, and each profession’s roles and responsibilities [73].

Building on this foundation, several actions are needed to support the effective and ethical integration of AITs into dementia care. Educational institutions should reinforce curricula in digital health literacy, algorithmic reasoning, and AI ethics across health care professions, including training on how to interpret algorithmic outputs and assess system reliability. In clinical settings, interprofessional training (through workshops, simulations, or case reviews) can foster shared understanding and critical reflection on AIT use [63]. Health care institutions should identify clinical champions to liaise with developers, support day-to-day integration, and promote peer learning [74]. Beyond health care and academic institutions, the development of these competencies should be supported by professional and regulatory bodies, including organizations issuing evidence-based practice guidelines, as well as geriatric and psychogeriatric associations, which can help contextualize AIT within ethical and clinical standards. Experts in long-term care can also contribute to preparing position statements and evaluation frameworks. From a research perspective, promoting nurse-led, co-designed research is essential to ensure that technological solutions are aligned with the day-to-day realities of care and person-centered values. Evidence shows that nurse-led research, especially by advanced practice nurses, improves care quality, successful implementation, and health system efficiency [75,76]. These efforts are particularly needed in settings where AITs are already being piloted, such as in long-term care and psychiatric units, to ensure safe, supported, and meaningful deployment from the outset.

Most of the studies retained used classic machine learning techniques, with only 2 exploring advanced methods like deep learning, which offers a greater capacity to model complex, temporal, and heterogeneous data [24,77]. Although some studies combined behavioral and physiological data, only 1 [55] applied deep learning to such multimodal inputs, suggesting that this is an underexplored area of research. The use of deep learning to explore the interactions between internal physiological states and external behavioral manifestations of the BPSD holds promise for enhancing the sensitivity, robustness, and contextual adaptability of detection systems. This is particularly relevant given the fluctuating and multifactorial nature of the BPSD. For instance, a review by Wu et al [78] showed that deep learning–based multimodal emotion recognition models outperformed conventional models by integrating behavioral, vocal, and physiological inputs, enabling a more robust and accurate interpretation of emotional and behavioral states.

However, the application of deep learning in health care raises well-known challenges, including a lack of transparency about analysis and prediction process, the limited interpretability of outputs, and a loss of clinical trust [24,74,79]. One promising strategy to address these concerns is the adoption of explainable AI approaches. These rely on techniques such as local interpretable model-agnostic explanations, which help clarify prediction accuracy, and Deep Learning Important FeaTures, which enable traceability by comparing neuron activations to reference states [24]. Promoting such approaches is essential to ensure that AI-driven decisions are not only technically reliable but also aligned with human values and ethical standards.

Furthermore, the practical value of advanced models depends not only on technical performance but also on feasibility and user acceptance. However, limited attention has been given to clinical and contextual factors, such as comorbidities, medication use, functional status, care environments, or staff ratios, which can all affect the expression and detection of the BPSD [2,79-82]. This reflects the exploratory nature of early-stage studies, which prioritized technical development, algorithmic performance, and system functionality. As previously highlighted, several studies also reported barriers to device acceptability, further underscoring the need for future research to consider issues involving clinical complexity and contextual variability.

In this regard, it is important to explore whether and how advanced methods, such as deep learning, can adapt to such variability and enhance the detection of BPSD. Multisite experimental studies should be conducted to assess the robustness and generalizability of algorithms across diverse care settings. Furthermore, the diversity of technological approaches used to detect BPSD highlights the need for experimental or quasi-experimental comparative studies to determine which AITs offer the most effective and reliable outcomes. Such evidence is critical for guiding clinical decision-making and future technological development based on proven efficacy.

In addition, mixed methods studies incorporating user-centered evaluations (such as perceived usefulness, usability, workflow integration, perceived technical performance, and interprofessional communication) and guided by implementation frameworks may offer valuable insights into how contextual factors shape technical outcomes and clinical integration while helping to anticipate potential barriers to adoption. Finally, longitudinal studies will be essential to assess AITs’ long-term impact on the detection and management of the BPSD, on the quality of life of people with dementia and their informal caregivers, on health care professionals’ workloads, and on overall cost efficiency.

### Limitations

This review had some limitations. The number of relevant publications was relatively limited compared with other reviews on the use of AITs in dementia care [34,36-38], which is probably attributable to AI’s relatively recent integration into the management of the BPSD. During our study screening process, a substantial number of publications referenced the use of AITs to detect dementia and evaluate functional status. However, some studies may have involved the BPSD without using this explicit terminology, which could have resulted in their exclusion during the selection of references. Also, no formal quality appraisal of the studies included was performed, as per standard scoping review methodology. However, eligibility criteria and dual screening helped ensure relevance. Despite addressing several dimensions of this topic, the overall lack of available data limited our exploration of other important contextual factors, such as costs, the availability of AITs, and health care infrastructure. This, too, restricted the scope of our recommendations.

### Conclusion

The use of AITs for the early detection of BPSD represents a rapidly emerging field of research with the potential to significantly enhance the quality of dementia care. This scoping review highlighted the diversity of AITs explored across various care settings, reflecting promising new avenues for improving the detection of BPSD. However, their clinical efficacy remains to be demonstrated. Future research should evaluate whether AITs support earlier and more accurate detection of BPSD, reduce symptom severity or duration, and improve the quality of life of people with dementia and their informal caregivers. Studies should also examine AITs’ impacts on clinical decision-making, care coordination, and workload management—across diverse populations and care contexts—to ensure relevance and generalizability.

To support their meaningful integration, nurses must be more actively involved in developing and evaluating these technologies. Their clinical expertise and proximity to patients make them key stakeholders in assessing the applicability of these tools in clinical settings. Their contribution should be strengthened through participatory design approaches, inclusion in pilot implementation studies, and interprofessional collaboration throughout the development cycles of new AITs.

The successful integration of AITs into dementia care will depend not only on technical innovation but also on inclusive, clinical, context-sensitive research strategies that can provide robust evidence of their impact in everyday practice.

## Supplementary material

10.2196/76074 Multimedia Appendix 1 Search strategies.

10.2196/76074 Checklist 1 PRISMA-ScR (Preferred Reporting Items for Systematic Reviews and Meta-Analyses extension for Scoping Reviews) fillable checklist.

## Abbreviations

AI: artificial intelligence
AIT: artificial intelligence–based technology
BPSD: behavioral and psychological symptoms of dementia
PRISMA-ScR: Preferred Reporting Items for Systematic Reviews and Meta-Analyses extension for Scoping Reviews
