# Supplementary material for: Using Artificial Intelligence–Based Technologies for the Early Detection of Behavioral and Psychological Symptoms of Dementia: Scoping Review
Source: JMIR Aging. 2025 Oct 21;8:e76074. doi: 10.2196/76074 (PMC12539798; doi:10.2196/76074)
Supplement: Multimedia Appendix 1 [file aging-v8-e76074-s001.pdf]

Multimedia Appendix 1: Search strategies

Bibliographic database search strategies

Joëlle Rosselet Amoussou, Medical Library-Cery, Lausanne University Hospital and University of Lausanne, Site de Cery, 1008 Prilly, Switzerland, ORCID 0000-0001-6871-5350

The research strategies were peer reviewed by another information specialist prior to execution.

Medline ALL Ovid

1946 to March 07, 2025

(exp "Aged"/ OR "Geriatric Nursing"/ OR "Geriatrics"/ OR Homes for the Aged/ OR (elder* OR eldest OR geriatr* OR "old age*" OR (older ADJ1 (patient* OR people OR subject* OR age* OR adult* OR man OR men OR woman OR women OR population* OR person*)) OR aging OR ageing OR senior* OR "late life" OR "oldest old*" OR "very old*" OR "home for the aged" OR "homes for the aged" OR geronto* OR psychoger*).ab,ti,kf,jw.) AND (exp Artificial Intelligence/ OR ((artificial ADJ3 intelligence) OR (machine* ADJ1 (intelligence OR learning)) OR "Computational Intelligence" OR "Computer Reasoning" OR "expert system*" OR robotic* OR "deep learning" OR "Computer heuristics" OR Hyperheuristics OR Metaheuristics OR "Automated reasoning" OR "Ambient intelligence" OR "fuzzy logic" OR (augmented ADJ3 (intelligence OR system*)) OR "intelligent agent*" OR "affective computing" OR "AI technique*").ab,ti,kf.) AND (exp Dementia/ OR Cognition Disorders/ OR (Dementia* OR alzheimer* OR (cognitive ADJ1 (defect* OR deficit OR disab* OR disorder* OR dysfunct* OR impair*)) OR "cognition disorder*").ab,ti,kf.) AND (Behavior/ OR exp Behavioral Symptoms/ OR exp Motor Activity/ OR exp Sexual Behavior/ OR exp Social Behavior/ OR exp Verbal Behavior/ OR Apathy/ OR Anxiety/ OR exp Sleep Wake Disorders/ OR Psychotic Disorders/ OR exp mood disorders/ OR ((behav* ADJ3 (abnormal OR aberr* OR crisis OR disturb* OR deviant OR manifestation* OR disorder* OR difficult* OR agitat* OR symptom* OR patholog* OR problem* OR assess* OR challenging OR change* OR defence OR defense OR psychosexual)) OR Problembehav* OR agitation OR "psychological symptom*" OR "neuropsychiatric symptom*" OR aggressi* OR antisocial OR asocial OR anti-social OR violence* OR violent OR defensive* OR sexual OR (social ADJ1 (activit* OR behav* OR contact*)) OR (verbal ADJ1 (behav* OR aggress*)) OR speech OR wandering OR "repetitive behav*" OR Sundowning OR Restlessness OR "altered mood" OR "mood change*" OR (mood ADJ1 (disorder* OR disturb*)) OR depressive OR depression OR "affective disorder*" OR apathy OR anxiety OR (sleep ADJ3 (difficult* OR disorder* OR disturb* OR problem* OR perturb*)) OR sleeplessness OR insomnia* OR misidentif* OR psychosis OR delusion* OR hallucination* OR ((motor OR psychomotor) ADJ activit*)).ab,ti,kf.)

475 results on 10.03.2025

Embase.com

('aged'/exp OR 'elderly care'/de OR 'geriatric care'/exp OR 'geriatric patient'/de OR 'geriatrics'/exp OR 'home for the aged'/exp OR (elder* OR eldest OR geriatr* OR "old age*" OR (older NEXT/1 (patient*

OR people OR subject* OR age* OR adult* OR man OR men OR woman OR women OR population* OR person*)) OR aging OR ageing OR senior* OR "late life" OR "oldest old*" OR "very old*" OR "home for the aged" OR "homes for the aged" OR geronto* OR psychoger*):ab,ti,kw,jt) AND ('artificial intelligence'/exp OR 'machine learning'/exp OR ((artificial NEXT/3 intelligence) OR (machine* NEAR/1 (intelligence OR learning)) OR "Computational Intelligence" OR "Computer Reasoning" OR "expert system*" OR robotic* OR "deep learning" OR "Computer heuristics" OR Hyperheuristics OR Metaheuristics OR "Automated reasoning" OR "Ambient intelligence" OR "fuzzy logic" OR (augmented NEAR/3 (intelligence OR system*)) OR "intelligent agent*" OR "affective computing" OR "AI technique*"):ab,ti,kw) AND ('cognitive defect'/de OR 'dementia'/exp OR (Dementia* OR alzheimer* OR (cognitive NEXT/1 (defect* OR deficit OR disab* OR disorder* OR dysfunct* OR impair*)) OR "cognition disorder*"):ab,ti,kw) AND ('behavior disorder'/exp OR 'agitation'/exp OR 'behavior'/de OR 'aggression'/exp OR 'antisocial behavior'/exp OR 'behavior change'/exp OR 'defensive behavior'/exp OR 'motor activity'/exp OR 'sexual behavior'/exp OR 'social behavior'/exp OR 'verbal behavior'/exp OR 'speech analysis'/exp OR 'wandering behavior'/exp OR 'behavior assessment'/exp OR 'restlessness'/exp OR 'mood change'/exp OR 'apathy'/exp OR 'anxiety'/exp OR 'sleep disorder'/exp OR 'psychosis'/exp OR 'mood disorder'/exp OR ((behav* NEAR/3 (abnormal OR aberr* OR crisis OR disturb* OR deviant OR manifestation* OR disorder* OR difficult* OR agitat* OR symptom* OR patholog* OR problem* OR assess* OR challenging OR change* OR defence OR defense OR psychosexual)) OR Problembehav* OR agitation OR "psychological symptom*" OR "neuropsychiatric symptom*" OR aggressi* OR antisocial OR asocial OR anti-social OR violence* OR violent OR defensive* OR sexual OR (social NEXT/1 (activit* OR behav* OR contact*)) OR (verbal NEXT/1 (behav* OR aggress*)) OR speech OR wandering OR "repetitive behav*" OR Sundowning OR Restlessness OR "altered mood" OR "mood change*" OR (mood NEXT/1 (disorder* OR disturb*)) OR depressive OR depression OR "affective disorder*" OR apathy OR anxiety OR (sleep NEAR/3 (difficult* OR disorder* OR disturb* OR problem* OR perturb*)) OR sleeplessness OR insomnia* OR misidentif* OR psychosis OR delusion* OR hallucination* OR ((motor OR psychomotor) NEXT/1 activit*)):ab,ti,kw) NOT ([conference abstract]/lim OR [conference paper]/lim)

1565 results on 10.03.2025

CINAHL EBSCO

(MH "Aged+" OR MH "Gerontologic Care" OR MH "Geriatrics" OR MH "Gerontologic Nursing+" OR (elder* OR eldest OR geriatr* OR "old age*" OR (older W0 (patient* OR people OR subject* OR age* OR adult* OR man OR men OR woman OR women OR population* OR person*)) OR aging OR ageing OR senior* OR "late life" OR "oldest old*" OR "very old*" OR "home for the aged" OR "homes for the aged" OR geronto* OR psychoger*)) AND (MH "Artificial Intelligence+" OR ((artificial W2 intelligence) OR (machine* N0 (intelligence OR learning)) OR "Computational Intelligence" OR "Computer Reasoning" OR "expert system*" OR robotic* OR "deep learning" OR "Computer heuristics" OR Hyperheuristics OR Metaheuristics OR "Automated reasoning" OR "Ambient intelligence" OR "fuzzy logic" OR (augmented N2 (intelligence OR system*)) OR "intelligent agent*" OR "affective computing" OR "AI technique*")) AND (MH "Dementia+" OR MH "Cognition Disorders" OR (Dementia* OR alzheimer* OR (cognitive W0 (defect* OR deficit OR disab* OR disorder* OR dysfunct* OR impair*)) OR "cognition disorder*")) AND (MH "Behavioral Symptoms+" OR MH "Behavior" OR MH "Behavioral

Changes" OR MH "Psychomotor Agitation" OR MH "Motor Activity+" OR MH "Sexual Behavior+" OR MH "Social Behavior+" OR MH "Verbal Behavior" OR MH "Wandering Behavior" OR MH "Apathy" OR MH "Anxiety" OR MH "Sleep Disorders+" OR MH "Psychotic Disorders+" OR MH "Affective Disorders" OR MH "Depression+" OR ((behav* N2 (abnormal OR aberr* OR crisis OR disturb* OR deviant OR manifestation* OR disorder* OR difficult* OR agitat* OR symptom* OR patholog* OR problem* OR assess* OR challenging OR change* OR defence OR defense OR psychosexual)) OR Problembehav* OR agitation OR "psychological symptom*" OR "neuropsychiatric symptom*" OR aggressi* OR antisocial OR asocial OR anti-social OR violence* OR violent OR defensive* OR sexual OR (social W0 (activit* OR behav* OR contact*)) OR (verbal W0 (behav* OR aggress*)) OR speech OR wandering OR "repetitive behav*" OR Sundowning OR Restlessness OR "altered mood" OR "mood change*" OR (mood W0 (disorder* OR disturb*)) OR depressive OR depression OR "affective disorder*" OR apathy OR anxiety OR (sleep N2 (difficult* OR disorder* OR disturb* OR problem* OR perturb*)) OR sleeplessness OR insomnia* OR misidentif* OR psychosis OR delusion* OR hallucination* OR ((motor OR psychomotor) W0 activit*)))

595 results on 10.03.2025

APA PsycInfo Ovid

1806 to March 2025 Week 1

(geriatric patients/ OR exp geriatrics/ OR exp aging/ OR older adulthood/ OR (elder* OR eldest OR geriatr* OR "old age*" OR (older ADJ1 (patient* OR people OR subject* OR age* OR adult* OR man OR men OR woman OR women OR population* OR person*)) OR aging OR ageing OR senior* OR "late life" OR "oldest old*" OR "very old*" OR "home for the aged" OR "homes for the aged" OR geronto* OR psychoger*).mp.) AND (exp artificial intelligence/ OR ((artificial ADJ3 intelligence) OR (machine* ADJ1 (intelligence OR learning)) OR "Computational Intelligence" OR "Computer Reasoning" OR "expert system*" OR robotic* OR "deep learning" OR "Computer heuristics" OR Hyperheuristics OR Metaheuristics OR "Automated reasoning" OR "Ambient intelligence" OR "fuzzy logic" OR (augmented ADJ3 (intelligence OR system*)) OR "intelligent agent*" OR "affective computing" OR "AI technique*").mp.) AND (exp dementia/ OR cognitive impairment/ OR (Dementia* OR alzheimer* OR (cognitive ADJ1 (defect* OR deficit OR disab* OR disorder* OR dysfunct* OR impair*)) OR "cognition disorder*").mp.) AND (exp behavior disorders/ OR agitation/ OR behavior/ OR exp behavior change/ OR exp social behavior/ OR wandering behavior/ OR exp behavioral assessment/ OR exp antisocial behavior/ OR exp behavior problems/ OR exp psychosexual behavior/ OR apathy/ OR restlessness/ OR exp anxiety/ OR exp sleep wake disorders/ OR exp psychosis/ OR exp affective disorders/ OR ((behav* ADJ3 (abnormal OR aberr* OR crisis OR disturb* OR deviant OR manifestation* OR disorder* OR difficult* OR agitat* OR symptom* OR patholog* OR problem* OR assess* OR challenging OR change* OR defence OR defense OR psychosexual)) OR Problembehav* OR agitation OR "psychological symptom*" OR "neuropsychiatric symptom*" OR aggressi* OR antisocial OR asocial OR anti-social OR violence* OR violent OR defensive* OR sexual OR (social ADJ1 (activit* OR behav* OR contact*)) OR (verbal ADJ1 (behav* OR aggress*)) OR speech OR wandering OR "repetitive behav*" OR Sundowning OR Restlessness OR "altered mood" OR "mood change*" OR (mood ADJ1 (disorder* OR disturb*)) OR depressive OR depression OR "affective disorder*" OR apathy OR anxiety OR (sleep ADJ3 (difficult*

OR disorder* OR disturb* OR problem* OR perturb*)) OR sleeplessness OR insomnia* OR misidentif* OR psychosis OR delusion* OR hallucination* OR ((motor OR psychomotor) ADJ activit*)).mp.)

370 results on 10.03.2025

Cochrane Database of Systematic Reviews Wiley

Issue 3 of 12, March 2025

(elder* OR eldest OR geriatr* OR (old NEXT age*) OR (older NEXT/1 (patient* OR people OR subject* OR age* OR adult* OR man OR men OR woman OR women OR population* OR person*)) OR aging OR ageing OR senior* OR "late life" OR (oldest NEXT old*) OR (very NEXT old*) OR "home for the aged" OR "homes for the aged" OR geronto* OR psychoger*):ab,ti,kw AND ((artificial NEXT/3 intelligence) OR (machine* NEAR/1 (intelligence OR learning)) OR "Computational Intelligence" OR "Computer Reasoning" OR (expert NEXT system*) OR robotic* OR "deep learning" OR "Computer heuristics" OR Hyperheuristics OR Metaheuristics OR "Automated reasoning" OR "Ambient intelligence" OR "fuzzy logic" OR (augmented NEAR/3 (intelligence OR system*)) OR (intelligent NEXT agent*) OR "affective computing" OR (AI NEXT technique*)):ab,ti,kw AND (Dementia* OR alzheimer* OR (cognitive NEXT/1 (defect* OR deficit OR disab* OR disorder* OR dysfunct* OR impair*)) OR (cognition NEXT disorder*)):ab,ti,kw AND ((behav* NEAR/3 (abnormal OR aberr* OR crisis OR disturb* OR deviant OR manifestation* OR disorder* OR difficult* OR agitat* OR symptom* OR patholog* OR problem* OR assess* OR challenging OR change* OR defence OR defense OR psychosexual)) OR Problembehav* OR agitation OR (psychological NEXT symptom*) OR (neuropsychiatric NEXT symptom*) OR aggressi* OR antisocial OR asocial OR anti-social OR violence* OR violent OR defensive* OR sexual OR (social NEXT/1 (activit* OR behav* OR contact*)) OR (verbal NEXT/1 (behav* OR aggress*)) OR speech OR wandering OR (repetitive NEXT behav*) OR Sundowning OR Restlessness OR "altered mood" OR (mood NEXT change*) OR (mood NEXT/1 (disorder* OR disturb*)) OR depressive OR depression OR (affective NEXT disorder*) OR apathy OR anxiety OR (sleep NEAR/3 (difficult* OR disorder* OR disturb* OR problem* OR perturb*)) OR sleeplessness OR insomnia* OR misidentif* OR psychosis OR delusion* OR hallucination* OR ((motor OR psychomotor) NEXT/1 activit*)):ab,ti,kw

0 results on 10.03.2025

Cochrane Central Register of Controlled Trials Wiley

Issue 2 of 12, February 2025

(elder* OR eldest OR geriatr* OR (old NEXT age*) OR (older NEXT/1 (patient* OR people OR subject* OR age* OR adult* OR man OR men OR woman OR women OR population* OR person*)) OR aging OR ageing OR senior* OR "late life" OR (oldest NEXT old*) OR (very NEXT old*) OR "home for the aged" OR "homes for the aged" OR geronto* OR psychoger*):ab,ti,kw AND ((artificial NEXT/3 intelligence) OR (machine* NEAR/1 (intelligence OR learning)) OR "Computational Intelligence" OR "Computer Reasoning" OR (expert NEXT system*) OR robotic* OR "deep learning" OR "Computer heuristics" OR Hyperheuristics OR Metaheuristics OR "Automated reasoning" OR "Ambient intelligence" OR "fuzzy logic" OR (augmented NEAR/3 (intelligence OR system*)) OR (intelligent NEXT agent*) OR "affective

computing" OR (AI NEXT technique*)):ab,ti,kw AND (Dementia* OR alzheimer* OR (cognitive NEXT/1 (defect* OR deficit OR disab* OR disorder* OR dysfunct* OR impair*)) OR (cognition NEXT disorder*)):ab,ti,kw AND ((behav* NEAR/3 (abnormal OR aberr* OR crisis OR disturb* OR deviant OR manifestation* OR disorder* OR difficult* OR agitat* OR symptom* OR patholog* OR problem* OR assess* OR challenging OR change* OR defence OR defense OR psychosexual)) OR Problembehav* OR agitation OR (psychological NEXT symptom*) OR (neuropsychiatric NEXT symptom*) OR aggressi* OR antisocial OR asocial OR anti-social OR violence* OR violent OR defensive* OR sexual OR (social NEXT/1 (activit* OR behav* OR contact*)) OR (verbal NEXT/1 (behav* OR aggress*)) OR speech OR wandering OR (repetitive NEXT behav*) OR Sundowning OR Restlessness OR "altered mood" OR (mood NEXT change*) OR (mood NEXT/1 (disorder* OR disturb*)) OR depressive OR depression OR (affective NEXT disorder*) OR apathy OR anxiety OR (sleep NEAR/3 (difficult* OR disorder* OR disturb* OR problem* OR perturb*)) OR sleeplessness OR insomnia* OR misidentif* OR psychosis OR delusion* OR hallucination* OR ((motor OR psychomotor) NEXT/1 activit*)):ab,ti,kw

66 results on 10.03.2025

Web of Science Core collection

Science Citation Index Expanded (1900-present), Social Sciences Citation Index (1900-present), Arts & Humanities Citation Index (1975-present), Conference Proceedings Citation Index-Science (1990-present), Book Citation Index (2005-present), Emerging Sources Citation Index (2005-present), Current Chemical Reactions and Index Chemicus

Advanced search > More options > Exact search

TS=((elder* OR eldest OR geriatr* OR "old age*" OR (older NEAR/0 (patient* OR people OR subject* OR age* OR adult* OR man OR men OR woman OR women OR population* OR person*)) OR aging OR ageing OR senior* OR "late life" OR "oldest old*" OR "very old*" OR "home for the aged" OR "homes for the aged" OR geronto* OR psychoger*) AND ((artificial NEAR/2 intelligence) OR (machine* NEAR/0 (intelligence OR learning)) OR "Computational Intelligence" OR "Computer Reasoning" OR "expert system*" OR robotic* OR "deep learning" OR "Computer heuristics" OR Hyperheuristics OR Metaheuristics OR "Automated reasoning" OR "Ambient intelligence" OR "fuzzy logic" OR (augmented NEAR/2 (intelligence OR system*)) OR "intelligent agent*" OR "affective computing" OR "AI technique*") AND (Dementia* OR alzheimer* OR (cognitive NEAR/0 (defect* OR deficit OR disab* OR disorder* OR dysfunct* OR impair*)) OR "cognition disorder*") AND ((behav* NEAR/2 (abnormal OR aberr* OR crisis OR disturb* OR deviant OR manifestation* OR disorder* OR difficult* OR agitat* OR symptom* OR patholog* OR problem* OR assess* OR challenging OR change* OR defence OR defense OR psychosexual)) OR Problembehav* OR agitation OR "psychological symptom*" OR "neuropsychiatric symptom*" OR aggressi* OR antisocial OR asocial OR anti-social OR violence* OR violent OR defensive* OR sexual OR (social NEAR/0 (activit* OR behav* OR contact*)) OR (verbal NEAR/0 (behav* OR aggress*)) OR speech OR wandering OR "repetitive behav*" OR Sundowning OR Restlessness OR "altered mood" OR "mood change*" OR (mood NEAR/0 (disorder* OR disturb*)) OR depressive OR depression OR "affective disorder*" OR apathy OR anxiety OR (sleep NEAR/2 (difficult* OR disorder* OR disturb* OR problem* OR perturb*)) OR sleeplessness OR insomnia* OR misidentif* OR psychosis OR delusion* OR hallucination* OR ((motor OR psychomotor) NEAR/0 activit*))) NOT (DT=(Meeting Summary) OR DT=(Meeting Abstract) OR DT=(Meeting))

458 results on 10.03.2025

ProQuest Dissertations & Theses A&I

Doctoral dissertations only

noft((elder* OR eldest OR geriatr* OR "old age*" OR (older NEAR/0 (patient* OR people OR subject* OR age* OR adult* OR man OR men OR woman OR women OR population* OR person*)) OR aging OR ageing OR senior* OR "late life" OR "oldest old*" OR "very old*" OR "home for the aged" OR "homes for the aged" OR geronto* OR psychoger*) AND ((artificial NEAR/2 intelligence) OR (machine* NEAR/0 (intelligence OR learning)) OR "Computational Intelligence" OR "Computer Reasoning" OR "expert system*" OR robotic* OR "deep learning" OR "Computer heuristics" OR Hyperheuristics OR Metaheuristics OR "Automated reasoning" OR "Ambient intelligence" OR "fuzzy logic" OR (augmented NEAR/2 (intelligence OR system*)) OR "intelligent agent*" OR "affective computing" OR "AI technique*") AND (Dementia* OR alzheimer* OR (cognitive NEAR/0 (defect* OR deficit OR disab* OR disorder* OR dysfunct* OR impair*)) OR "cognition disorder*") AND ((behav* NEAR/2 (abnormal OR aberr* OR crisis OR disturb* OR deviant OR manifestation* OR disorder* OR difficult* OR agitat* OR symptom* OR patholog* OR problem* OR assess* OR challenging OR change* OR defence OR defense OR psychosexual)) OR Problembehav* OR agitation OR "psychological symptom*" OR "neuropsychiatric symptom*" OR aggressi* OR antisocial OR asocial OR anti-social OR violence* OR violent OR defensive* OR sexual OR (social NEAR/0 (activit* OR behav* OR contact*)) OR (verbal NEAR/0 (behav* OR aggress*)) OR speech OR wandering OR "repetitive behav*" OR Sundowning OR Restlessness OR "altered mood" OR "mood change*" OR (mood NEAR/0 (disorder* OR disturb*)) OR depressive OR depression OR "affective disorder*" OR apathy OR anxiety OR (sleep NEAR/2 (difficult* OR disorder* OR disturb* OR problem* OR perturb*)) OR sleeplessness OR insomnia* OR misidentif* OR psychosis OR delusion* OR hallucination* OR ((motor OR psychomotor) NEXT/0 activit*)))

33 results on 10.03.2025

Supplementary searches

Sofia Fernandes, School of Health Sciences, University of Applied Sciences and Arts Western Switzerland (HES-SO), Chemin de l'Agasse 5, CH-1950 Sion, Switzerland

Citationchaser

https://estech.shinyapps.io/citationchaser/

Haddaway, N. R., Grainger, M. J., Gray, C. T. (2021) citationchaser: An R package and Shiny app for forward and backward citations chasing in academic searching. doi: 10.5281/zenodo.4543513

A backward and Forward citation search was performed on the key articles using Citationchaser.
Copy/paste in *Article Input > Digital Object Identifiers (DOIs)*

10.3928/00989134-20220309-01, 10.1016/j.eclinm.2024.103032, 10.48550/arXiv.2110.09868, 10.3390/info14080433, 10.3233/JAD-181033, 10.1371/journal.pone.0195605,

10.3390/ijerph18052720, 10.5664/jcsm.11436, 10.1007/s12652-010-0043-x, 10.12968/nrec.2019.21.9.489, 10.1002/dad2.12305, 10.1016/j.irbm.2013.02.002

- Backward citation chasing: *323 references and 321 unique records after deduplication* (07.03.2025)
- Forward citation chasing : *Cited a total of 490 times. This corresponds to 477 unique article IDs.* (07.03.2025)

ACM Digital Library
https://dl.acm.org/

Advanced search

Title : dementia* OR alzheimer*
Anywhere : artificial intelligence OR AI

211 Results for: [[Title: dementia*] OR [Title: alzheimer*]] AND [[All: artificial intelligence] OR [All: ai]] (16.06.2025)
